# Profiling the Bacterial Diversity in a Typical Karst Tiankeng of China

**DOI:** 10.3390/biom9050187

**Published:** 2019-05-14

**Authors:** Gaozhong Pu, Yanna Lv, Lina Dong, Longwu Zhou, Kechao Huang, Danjuan Zeng, Ling Mo, Guangping Xu

**Affiliations:** 1 Guangxi Key Laboratory of Plant Conservation and Restoration Ecology in Karst Terrain, Guangxi Institute of Botany, Guangxi Zhuang Autonomous Region and Chinese Academy of Sciences, Guilin 541006, China; donglina@gxib.cn (L.D.); longzhouwu@163.com (L.Z.); huangkechao2011@163.com (K.H.); djzeng221@163.com (D.Z.); ml@gxib.cn (L.M.); xugpgx@163.com (G.X.); 2 School of Pharmacy and Biological Sciences, Weifang Medical University, Weifang 261053, China; lynlyna@163.com

**Keywords:** bacterial diversity, karst tiankeng, refugia, habitat heterogeneity, illumina sequencing

## Abstract

While karst tiankengs have a higher capacity to act as safe havens for biodiversity in changing climates, little is known about their soil microorganisms. To fill this gap, we investigate the distribution and driving factors of the bacterial community in karst tiankeng systems. There is a significant difference in the soil characteristics between the inside and the outside of a karst tiankeng. At the karst tiankeng considered in this study, the bacterial composition, in terms of the operational taxonomic unit (OTU), was found to be significantly different in different soil samples, taken from diverse sampling sites within the collapsed doline or the external area, and showed a high habitat heterogeneity. The dominant phylum abundances vary with the sampling sites and have their own indicator taxa from phylum to genus. Unlike the primary controlling factors of plant diversity, the microclimate (soil moisture and temperature), soil pH, and slope dominated the distribution of the bacterial community in karst tiankeng systems. Our results firstly showed the distribution characteristics of bacterial communities and then revealed the importance of microhabitats in predicting the microbial distribution in karst tiankeng systems.

## 1. Introduction

Ongoing anthropogenic disturbances (such as climate change) are considered to pose a potential threat to the distribution of plant species and ecosystems processes [1]. Due to their special environmental characteristics, refugia could provide stable habitats for plant species and protect them from the negative effects of environmental changes [2,3]. Previous studies have shown that refugia, especially karst tiankengs (a kind of important modern refugia) preserved more old-growth and endemic plant species than the degraded habitats outside tiankeng, and had a higher capacity to act as safe havens for biodiversity in changing climates [3,4,5]. Yet, limited information is available about the microbial communities, the soil, and the relationships between the below- and above-ground microbial-plant communities in these refugia (especially karst tiankengs).

Different from other landforms, karst landforms form many unique natural landscapes, such as fenglin, fengcong, cave systems, and karst tiankengs. Among them, the karst tiankeng, as a typical refugium, was first named by Zhu [6] in 2001 and is described as a large karst depression described as collapsed dolines that are more than about 100 m in width and depth [7]. Due to the trapping of surrounding rock walls and terrain constraints, the outside and inside habitats of tiankengs are independent of each other. For thousands of years, the inside habitats of tiankengs have formed a unique ecosystem, which is characterized by an original microclimate, unique hydrothermal conditions, and soil fertility [4,5,8]. As typical modern refugia, karst tiankengs preserve more old-growth and endemic plant species than the degraded habitats outside tiankengs, and have a higher capacity to act as safe havens for biodiversity in changing climates [4,5,8]. Currently, there are more than 200 karst tiankengs that have been discovered globally, with approximately 170 in China [4,9].

In recent decades, substantial research on karst tiankengs has been conducted [4,5,9,10,11,12,13,14,15]. However, these studies mainly focused on geology (the shape, formation, and evolution mechanism of karst tiankengs) [9,10,11], the diversity of animal and plant species [5,12,13], and the value of natural tourism resources and organic pollutants [15]. In particular, compared with the habitats outside tiankeng, habitats inside tiankengs possess higher floristic diversity, species richness, remarkably higher uniqueness, water-bearing capability, and endemic species [5]. Compared with plant communities outside tiankengs, plant communities inside tiankengs are more diverse, have a stronger uniqueness and originality, and have a higher habitat heterogeneity, lower similarity, and poor reproducibility [4,5]. These studies also found that human disturbances, topography characteristics, temperature, soil moisture, and the size of tiankengs have a greater impact on the vegetation distribution pattern inside tiankengs [16]. In particular, the bottoms of karst tiankengs have a lower temperature and less interference, but can receive more water and nutrients, resulting in a higher humidity, which will undoubtedly affect the composition and structure of the vegetation [17,18]. However, only a few studies have focused on the soil fungi that have been isolated from tiankeng soil [19,20], such as *Exophiala* sp., a dark septate endophytic fungi (DSE), which had a positive effect on improving the drought resistance and growth of *Zenia insignis* and *Caesalpinia sappan* [19], and *Trichoderma* sp., which had an enhanced effect on the degradation of feather proteins [20]. Nonetheless, there is limited knowledge concerning the distribution and contribution of soil microorganisms in karst tiankengs, although they play an important role in the cycling of carbon and nutrients in terrestrial habitats [21].

Soil bacteria are one of the most diverse and species-rich biota on the planet and are involved in nearly all of the important biogeochemical cycles in terrestrial ecosystems. Previous studies on soil microbes in different karst ecosystems, such as a typical karst forest, karst soils, and associated caves, have shown that diverse soil physicochemical properties (e.g., pH and Ca), sampling sites, and potential interactions between different taxa in the process of vegetation restoration may affect the bacterial community’s structure [22,23,24,25,26]. However, little is known about the bacterial community’s structure and compositions between karst tiankeng soils and the associated outside surface.

Here, we compared and characterized the soil bacterial diversity and community structure inside and outside the Shengmu tiankeng, a typical karst refugium, with a well-developed vegetation in China. To further explore the primary driving factors leading to the changes in soil bacteria, we investigated the plant species richness, soil physicochemical charactersistics, and temperatures inside and outside the Shengmu tiankeng. To answer the above questions, a high-throughput sequencing of the 16S rRNA gene was employed to determine the phylogenetic diversity and taxonomic richness, as well as associate these with measured environmental variables to reveal the driving factors for the bacterial distribution in the tiankeng.

## 2. Materials and Methods

### 2.1. Site Description

**Figure 1:**
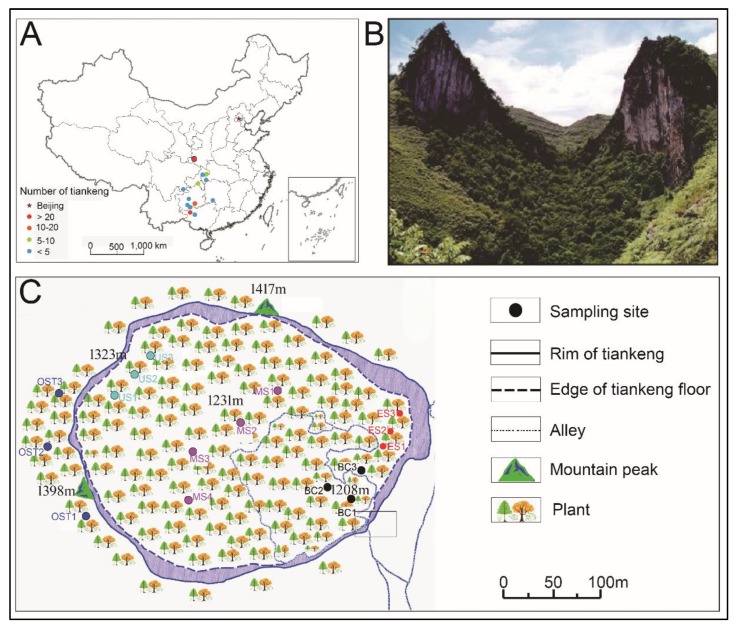
Location of the study site and tiankengs distribution in China (**A**). An aerial view of the Shenmu tiankeng ecosystem (**B**). The sampling locations outside and inside the Shenmu tiankeng are shown in (**C**). BC, the bottom central of the Shenmu Tiankeng; MS, the middle slope of the Shenmu Tiankeng; US, the upper slope of the Shenmu Tiankeng; ES, the east slope of the Shenmu Tiankeng; OST, the outside of the Shenmu tiankeng.

The Shenmu tiankeng (106°10′–106°51′ E, 24°30′–25°03′ N) is located in Tongle town, Guangxi Province, Southwestern China (Figure 1A,B). This area is located in the southeastern part of the Yunnan-Guizhou Plateau, which is characterized by the Fenglin and Fengcong karsts. The area belongs to a subtropical humid climate zone, with an annual precipitation of 1100–1500 mm, a relative humidity of 83%, and an annual average temperature of 16.5 °C [27]. The Shenmu tiankeng is 370 m long and 340 m wide, with an area of 70,860 m^2^ (Figure 1B). Its slope is steep, oriented from northwest to southeast, and its surface is filled with collapsed debris and covered by dense secondary forests. The dominant plant species are *Platycarya longipes*, *Acer tonkinense* subsp. *kwangsiense*, *Handeliodendron bodinieri*, *Machilus glaucifolia*, *Metapanax davidii*, *Elatostema brachyodontum*, and *Pilea glaberrima* Bl. [4,5,28].

### 2.2. Experimental Design

Previous studies indicated that the climate, soil, and floristic community characteristics also vary within a karst tiankeng or sinkhole [8,27,28]. The distribution pattern of the forest vegetation in karst tiankeng (including the Shenmu tiankeng) forest is, from the lower to the upper parts, followed by a lower subtropical evergreen broadleaf forest, a warm coniferous forest, an evergreen-deciduous broadleaf-mixed forest, and a warm coniferous forest [16]. Based on the soil and plant types inside the Shenmu tiankeng (Table A1), four different sites were selected (Figure 1C): the bottom central (BC), the middle slope (MS), the upper slope (US), and the east slope (ES), which is steeper and obviously different from other habitats inside tiankeng. In addition, a typical sporadic forest fragment outside of the Shenmu tiankeng (OST) was selected as a contrast (Figure 1C). An investigation and sampling were conducted at the five sampling sites, and each sampling site contained three contiguous 20 × 20 square meters (m^2^). At each quadrat that was representative of the typical local vegetation, all plants (including trees, shrubs, and herbs) were investigated and identified in terms of their species to calculate the Shannon–Wiener diversity index, which is one of several diversity indices used to measure diversity in categorical data and calculated following the methods of Lin [16]. Before collecting the soil samples, the litter layer was removed, soil samples were collected in an “S” shape, and five points were joined together to form a single sample. After removing the large pieces of debris, the fresh soil samples were sieved (2 mm sieve) and transported to the laboratory under −4 °C conditions. Samples for the physicochemical property analysis and DNA extraction were stored at −4 °C and −80 °C, respectively.

### 2.3. Soil Physicochemical Analyses

Soil pH values were measured using a pH-meter (ST3100, Ohaus Instruments, NJ, USA) at a ratio of 1:5 (mass/volume), after shaking equilibration for approximately 30 min. The determination of the total nitrogen (TN) and total organic carbon (TOC) were conducted according to standard procedures [29]. The soil total phosphorus (TP) was determined using ultraviolet spectrophotometry with the molybdate ascorbic acid method [30]. To determine the content of the soil trace (e.g., Cs, Pb, Cd, Ni, Cr, As, Hg, Be, Ho, Lu, Tm, W, Y, Rb, Sr, and Ba) and major elements (e.g., K, Ca, Fe, and Mg), 0.2 g of the soil samples were digested with 2 mL HCl, 8 mL HNO_3_, and 0.25 mL H_2_O_2_ at approximately 140 °C, 165 °C, and 190 °C for 10 min each, followed by 210 °C and 225 °C for 20 min each. The soil trace element (e.g., Cs, Be, Tm, and Lu) contents were measured using ICP-MS (NexION 350, PerkinElmer Instruments, Waltham, MA, USA), and the major element (e.g., K, Ca, Fe, and Mg) contents were measured using atomic absorption spectrometry (AAS-6800, Shimadzu, Kyoto, Japan).

### 2.4. Soil Bacterial Biodiversity

DNA was extracted in triplicate from 0.5 g of the soil samples using an E.Z.N.A.® soil DNA Kit (Omega Bio-tek, Norcross, GA, U.S.). The final DNA concentration was determined using a NanoDrop 2000 UV-vis spectrophotometer (Thermo Scientific, Wilmington, DE, USA), and the DNA quality was confirmed for the subsequent analysis of the microbial community by 1% agarose gel electrophoresis. The V3-V4 regions of the 16S rRNA gene were amplified with primers 806R (5ʹ-GGACTACHVGGGTWTCTAAT-3ʹ) and 338F (5ʹ- ACTCCTACGGGAGGCAGCAG-3ʹ) using a PCR system (GeneAmp 9700, ABI, Waltham, MA, USA), according to the program of 94 °C, for 3 min, followed by 5× (94 °C, 30 s; 45 °C, 20 s; 65 °C, 30 s) and 25× (94 °C, 20 s; 55 °C, 20 s; 72 °C, 30 s), and a final extension at 72 °C for 5 min. Purified amplicons were delivered to Sahnghai Majorbio Bio-Pharm Technology Co., Ltd. (Shanghai, China) for high-throughput pyrosequencing. Then, we sent the tested bacteria data to the Sequence Read Archive (SRA) database of the National Center for Biotechnology Information (NCBI) and obtained the accession numbers of SRP165142.

### 2.5. Statistical Analyses

The bacterial alpha diversity, including the Sobs and abundance-based coverage estimator (Ace) index, Chao richness, Shannon’s and Simpson’s diversity, was calculated using MOTHUR (version v.1.30.1) (Schloss team: http://www.mothur.org/, 2009) [31]. The bacterial beta diversity was calculated using principal coordinate analysis (PCoA) on the basis of the Braye Curtis results in QIIME [32]. The differences in the plant Shannon indices, soil characteristics, bacterial alpha diversity, quality sequences, and observed operational taxonomic unit (OTU) numbers were tested for significance using a one-way analysis of variance (ANOVA) (SPSS 22.0). To test the significant differences between the bacterial communities of the different sample sites, an analysis of similarity (ANOSIM) was carried out, based on the Bray–Curtis dissimilarities. To evaluate significant taxonomic differences between the five different sites, linear discriminant analysis effect size (LEfSe) was conducted with the MASS package in R. The selection of environmental factors was based on the functions of env-fit (permu = 999) and removed those factors with a vif value >10. To identify the relationship between the bacterial communities and environmental factors, a redundancy analysis (RDA) was carried out in R software (Version v.3.1.1, vegan package) (R Development Core Team: https://www.r-project.org/, 2016). To further understand the important role of the bacteria present in karst tiankengs, 16S rRNA based high-throughput sequencing data was performed by phylogenetic investigation of communities through the reconstruction of unobserved states (PICRUSt10) program, which were then analyzed in the context of the cluster of orthologous groups (COG) database [33]. All values are expressed as the mean ± standard error.

## 3. Results

### 3.1. Environmental Factor Characteristics

The environmental factor characteristics of the sample sites, from the inside and outside of the Shenmu tiankeng, are summarized in Table 1, Table A1, and Table A2. The sampling sites have a great impact on both the plant communities and soil characteristics. The plant Shannon–Wiener index was the highest in the middle slope (MS site), and the lowest in both the east slope (ES site), with a steep slope, and outside the Shenmu tiankeng (OST site) (*p* < 0.05; Table 1). As for the soil characteristics, the BC site has the lowest soil temperature of the sites (*p* < 0.05; Table 1). Compared with the steep slope site of the inside tiankeng (ES) and outside tiankeng sites (OST), from the small gradient slope sites (e.g., BC, MS, and US), the collected soil samples were characterized by black humus soil (Table A1), higher TOC, TN, and Ca concentrations, but lower concentrations of major elements (K, Mg, and Fe) (Table 1), and many traces (e.g., Be, Pb, Cd, Cr, As, Tm, and Lu) of element concentrations (Table A2). In particular, the concentration of some trace elements (such as Y, Ho, Tm, and Lu) in the sample OST was more than 32-fold higher than in the US samples and 10-fold higher than in both samples, BC and MS, while there were no significant differences in many trace elements (such as Be, Na, and W) between the ES site and OST site (*p* < 0.05, Table A2).

### 3.2. Bacterial Community Compositions inside and outside of Shenmu Tiankeng

**Figure A1:**
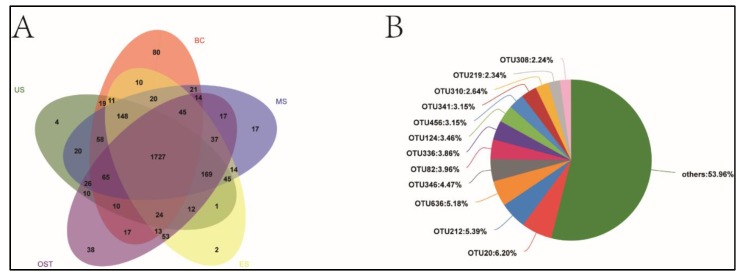
Venn diagram of OTU among different sampling sites (**A**) and the lower-central part of the Shenmu tiankeng (BC) site-specific OTU (**B**). MS: the middle slope of the Shenmu Tiankeng; US: the upper slope of the Shenmu Tiankeng; ES: the east slope of the Shenmu Tiankeng; OST: the outside of the Shenmu tiankeng. Others: 53.96% indicate the proportion of all the unidentified total OTUs.

The gene copy of bacteria varied from 1.34 × 10^7^ to 1.94 × 10^7^ g^−1^ dry soil across all samples. No sample-location-associated abundance pattern was observed. In total, we obtained about 5.99 × 10^5^ sequences, and each sample had 36,453–38,495 sequences (Table 2). The average read lengths were 440 bp. There were 2747 unique OTUs, which belonged to 39 bacterial Phylum. A Venn diagram demonstrated that a total of 1727 OTU were observed in all soil samples, and the site-specific OTU ranged from 2 (ES site) to 80 (BC site) (Figure A1A). The most abundant OTU of BC site-specific included OTU20 (6.2%; Gemmatimonadetes), OTU212 (5.4%; Nitrospira), and OTU636 (5.2%; Burkholderia-Paraburkholderia) (Figure A1B). The bacterial alpha diversity indicated that the Sobs, Ace, and Chao1 values for the MS samples were considerably higher than those for the ES and OST samples (Table 2). The Shannon’s diversity was higher, but the Simpson’s diversity was significantly lower for the BC, MS, and US samples than for the ES and OST samples (Table 2).

**Figure 2:**
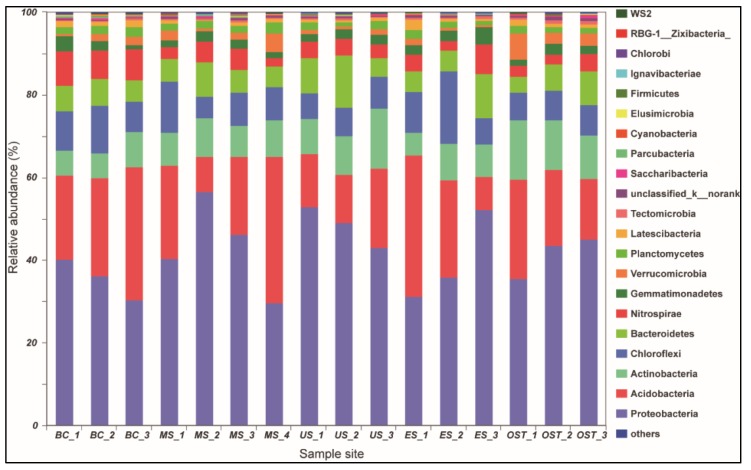
Relative abundance (%) of the dominant bacteria at the phylum level. Lanes BC1–BC3, MS1–MS4, US1–US3, ES1–ES3, and OST1–OST3 correspond to the five sampling sites of the lower-central area, the middle slope, the upper slope, the east slope, and the outside of the Shenmu tiankeng, respectively.

**Figure 3:**
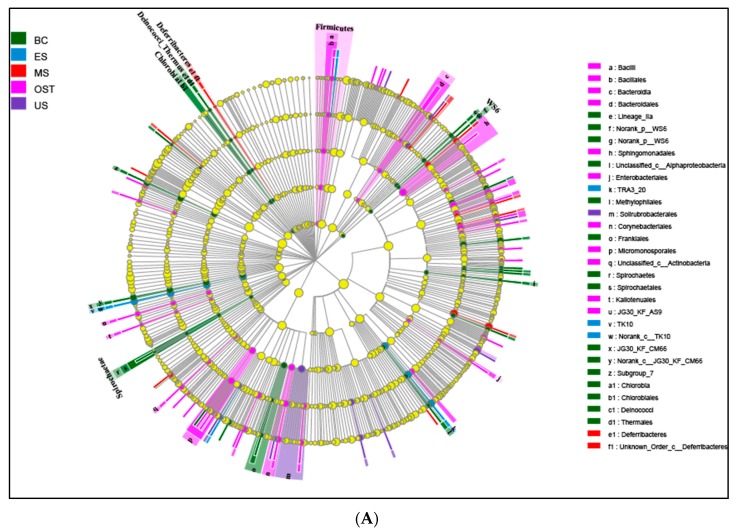

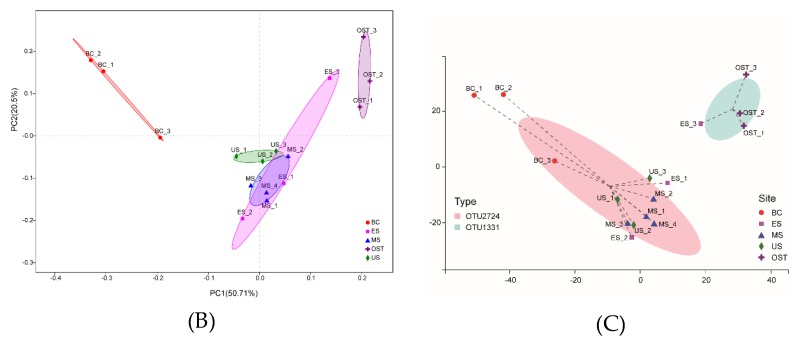
Least discriminant analysis (LDA) (**A**), principal coordinate analysis (PCoA) (**B**), and the enterotype analysis (**C**) of bacterial communities. A: Nodes from the outside to the inside circles show the different levels (from genus to phylum) of bacterial taxon; circles represent taxa that are abundant in the BC, ES, MS, OST, and US sampling sites, respectively; yellow nodes stand for taxa that are not significantly discriminate between habitats; the diameter of the node is positively correlated with the relative abundance of the taxon. The abbreviations for the samples are described in Figure 1.

At the phylum level, Proteobacteria and Acidobacteria, with relative abundances ranging from 30.33% to 56.67% and from 8.15% to 35.41%, respectively, were the most abundant groups across all samples (Figure 2). Furthermore, the relative abundances of Chloroflexi, Actinobacteria, Nitrospirae, and Bacteroidetes varied among the sampling sites. For example, Chloroflexi was more abundant in the US sample than in the other samples (14.07% versus 7.11–10.97%, respectively), as was Nitrospirae in the BC sample (8.43% versus 3.07–4.65%) and Actinobacteria in the OST sample (12.23% versus 6.08–10.43%). To evaluate the bacterial compositions and differentially dominant clades of diversity in the Shenmu tiankeng ecosystems, LEfSe was used to determine the differentially abundant taxa and evaluate the bacterial compositions at each taxonomic level for the different sites. The results showed that 142 differentially abundant taxa were carried out in all of the soil samples. In particular, 54 and 62 differentially abundant taxa from genus to phylum levels were detected in the BC and OST sites, respectively (Figure 3A). In addition, the MS sample, ES and OST samples, and US sample had their own indicator taxa from phylum to genus levels, from class to genus and from order to genus, respectively. In particular, Chlorobi, Spirochaetae, Deinococci-Thermus, and WS6 were specific to the BC site, Deferribacteres, Microgenomates, and BJ-169 were specific to the MS site, while Firmicutes and WWE3 were specific to the OST site. The ANOSIM analysis showed that the bacterial community compositions at the OTU level were significantly different among the five samples (R = 0.63, *p* = 0.001). To better visualize and explore the obtained data, principal coordinates analysis (PCoA) was performed (Figure 3B). The first two principal components can explain 71.2% of the variance in the microbial community’s composition. The bacterial community’s structure indicated that the five sample sites (except for the ES site) were well-separated from each other. Meanwhile, PCoA plots also showed comparatively high discrepancies between the upper site (ES–3) and both the middle (ES–2) and lower (ES–1) sites of ES, such as ES–3, which was grouped closer together with the OST samples, suggesting that there is a higher similarity of bacterial communities in these four sites. The enterotype analysis also indicated that all samples from the Shenmu tiankeng (except ES–3) belonged to the OTU2734 type (Halieaceae), while the ES–3 and all the outside sites strongly belonged to the OTU1331 (Desulfurellaceae) type (Figure 3C).

### 3.3. Driving Factors of Bacterial Distribution

**Figure 4:**
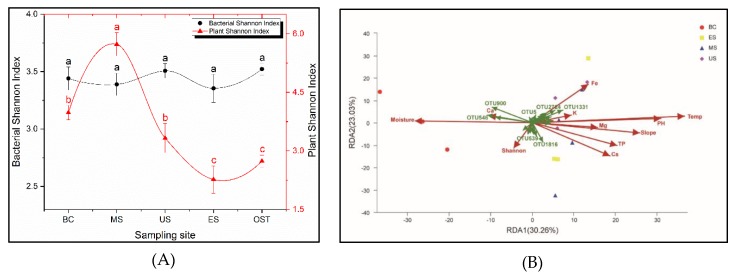
Changes of the plant and bacteria (Shannon index) between outside and inside-tiankeng (**A**) and Redundancy analysis (RDA) among the environmental variables and plant and the bacterial community compositions from the Shenmu tiankeng samples at the operational taxonomic unit (OTU) level (**B**). Sample abbreviations are described in Figure 1. TP, total phosphorus; TOC, total organic carbon; Temp., soil temperature; Shan., plant Shannon–Wiener; Cs, Caesium; K, Potassium; Mg, Magnesium; Ca, Calcium; Fe, Ferrum.

**Figure 5:**
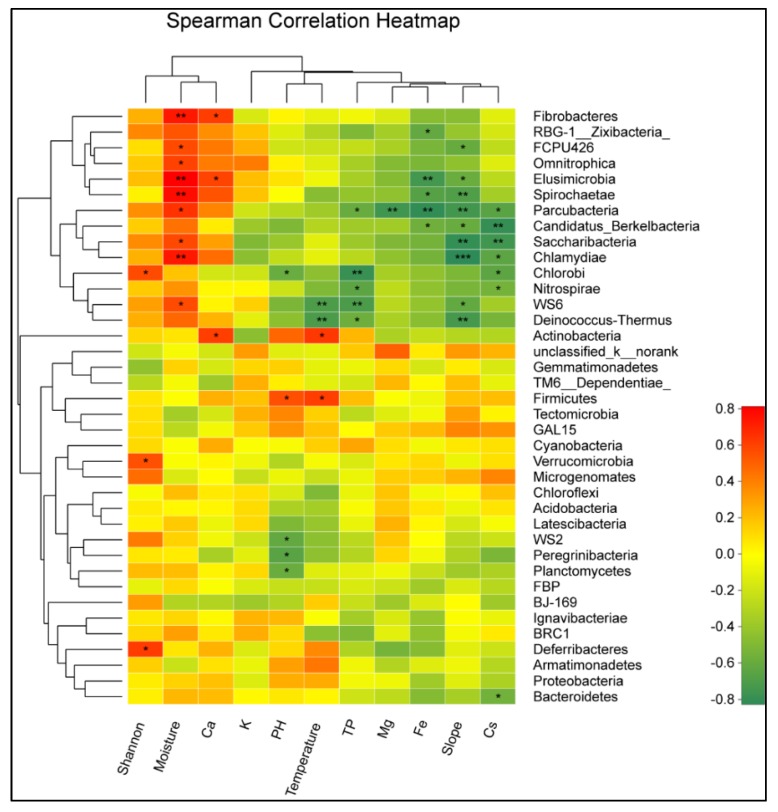
Heatmap of the 38 most abundant phylum of the bacteria in the samples collected from different sites. The heatmap colors represent the relative percentages of the microbial phylum assignments within each sample. One star shows 0.01 < *p* ≤ 0.05, a double star shows 0.001 < *p* ≤ 0.01, and a triple star shows *p* ≤ 0.001. Abbreviations are described in Figure 4.

The sample sites have a significant impact on the plant diversity, but have no effect on the bacterial alpha diversity (Figure 4A). The plant diversity was higher in the MS site and was lower in the ES and OST sites, ranging from 2.2 to 5.7. Redundancy analysis (RDA) was performed for determining the correlation between the plant diversity, soil physicochemical properties, and the bacterial community’s composition of the Shenmu tiankeng ecosystem. The results at the OTU level showed that the first two axes of RDA account for 30.26% and 23.03% of the total data variation, respectively, of the total variation in the data (Figure 4B). There were positive correlations between the plant Shannon–Wiener, Ca, and moisture and between the soil temperature, pH, slope, Fe, TP, Cs, Mg, and K (Figure 4B). Among those investigated, the moisture, soil temperature, slope, and pH are key factors associated with the bacterial community compositions at the OTU level (Table A3; *p* < 0.05). Meanwhile, the relationship between the environmental factors and bacterial phylum was different (Figure 5). The relative abundance of WS6, Parcubacteria, Chlamydiae, Saccharibacteria, Fibrobacteres, Elusimicrobia, Spirochaetae, FCPU426, and Omnitrophica was negatively correlated with the soil temperature and TP but positively correlated with the soil moisture; Chlorobi, WS2, Peregrinibacteria, and Planctomycetes were negatively correlated with the soil pH, while Firmicutes was positively correlated with the soil moisture; Parcubacteria was negatively correlated with TP, Mg, Fe, the slope, and Cs; Chlammdiae and Saccharibacteria were negatively correlated with slope and Cs; and Elusimicrobia and Spirochaetae were negatively correlated with slope and Fe (Figure 5).

### 3.4. The Bacterial Functional Genes

**Figure A2:**
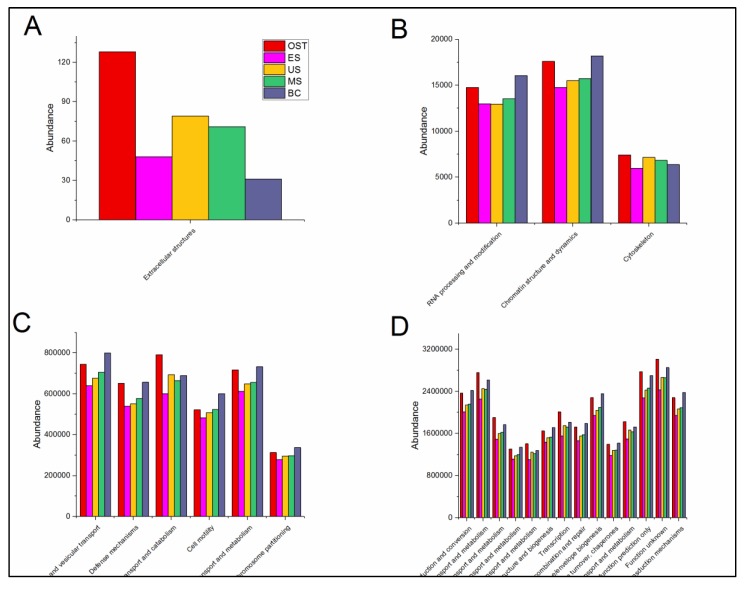
Predictive analysis of bacterial COG genes in different samples under different abundance values: A, abundance value < 5000; B, 5000 ≤ abundance value < 20,000; C, 20,000 ≤ abundance value < 8,000,000; D, 8,000,000 ≤ abundance value. Abbreviations are described in Figure A1.

**Figure A3:**
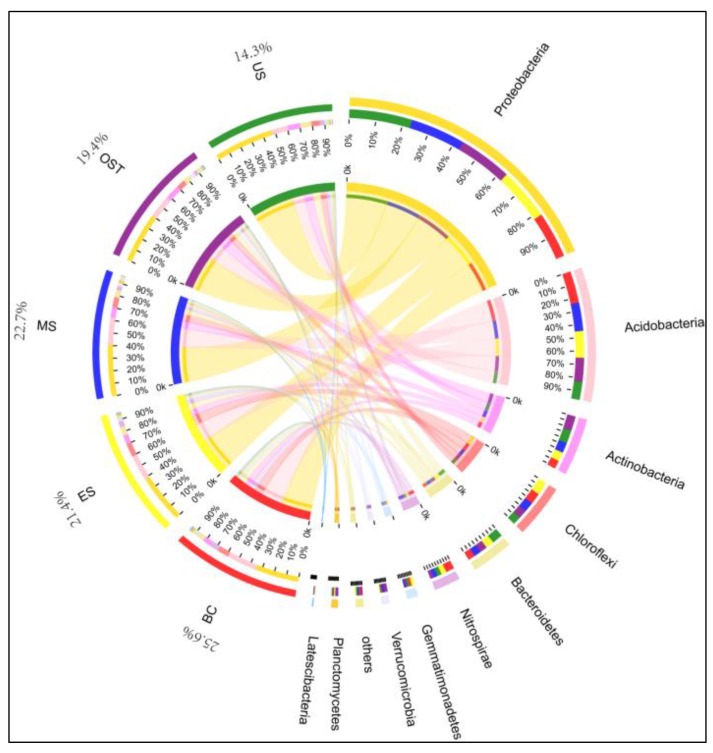
Circos diagram of the bacterial communities for each site at the phylum level. The percentage indicates the abundance of Acidobacteria in different sample sites. Abbreviations are described in Figure A1.

Based on the COG analysis, we describe the soil microbial functional genes (Figure A2 and Figure A3). In general, all of the categories related to the core metabolic functions (e.g., especially extracellular structures and RNA processing and modification, energy production and conversion, and general function prediction only) indicated a relatively higher abundance on the outside of the Shenmu tiankeng than in the upper (MS and US) or edge (ES) sites of the Shenmu tiankeng. However, some of the categories (e.g., the cell motility, energy production and conversion, and general function prediction) showed a relatively higher abundance at the bottom of the Shenmu tiankeng than on the outside of the Shenmu tiankeng.

## 4. Discussion

Previous studies showed that refugia, such as karst tiankengs, can form under the predicted anthropogenic climate change, and they can thus be a priority for conservation, because they can promote the survival of biota under adverse conditions [2,3,5]. In particular, compared with the arid, degraded landscapes on the outside of tiankengs, the bottoms of the inside of tiankengs can obtain more water and nutrients, resulting in a higher humidity, soil moisture, and plant biodiversity in changing climates [3,5,17,18]. Our research on plant diversity inside and outside of the Shenmu tiankeng was consistent with the above results. In addition, our results also showed that there were significantly higher TOC, TN, and Ca, but some major (K, Mg, and Fe; Table 1) and trace (e.g., Be, Tm and Lu, Table A1) elements were lower in the samples of the inside of the Shengmu tiankeng (except for the ES site) than in that from the outside of the Shengmu tiankeng samples, agreeing with a previous study, in which the content of Pb, Cd, Ni, Cr, As, and Hg in the top soil (outside-tiankeng) of the Dashiwei tiankeng was found to be significantly higher than that at the bottom [15]. This may be attributed to the origin of the soil. Our results showed that the sampling soils, collected from the steep slope of the Shenmu tiankeng (ES) and outside of the Shenmu tiankeng (OST), were mainly characterized by a light black powdery clay soil, little humus, and similar concentrations of TOC, TN, and many elements, suggesting that they have the same origin. The possible reason may be that the soil of the ES site was formed when the soil from the outside of the tiankeng fell, which occurred relatively recently. Furthermore, the steeper slopes also made it difficult for litters to accumulate in the ES site. However, the other sampling soils collected from the inside of the tiankeng were mainly characterized by black humus and more organic matter. This may have contributed to its higher plant species richness, which provided more litter. It is well-known that the formation of the soil in karst areas is the result of carbonate rocks being subjected to karst corrosion weathering and the accumulation of living beings over a long time. During its development, compared with the outside soil, the soil on the inside of the tiankeng could receive more litter and less residue formed after the weathering of carbonate rocks, resulting in a higher content of TOC, TP, and TN, but a lower content of many metal elements in these soils. As a result, the soil inside and outside of the tiankeng showed a great difference, which may have a great impact on biodiversity.

Karst tiankengs, as typical refugia, have preserved more old-growth and endemic plant species than the degraded habitats outside of the tiankeng [3,5]. Previous studies showed that there was a remarkably higher taxonomic richness on the inside than on the outside of the tiankeng habitats, from the species to the familial levels [5,16]. In addition, heterogeneity in plant composition, from the species to the familial levels, is significant throughout the habitats [5]. This suggests that heterogeneity may be an important environmental factor in karst tiankeng ecosystems. However, there is limited knowledge concerning the distribution and contribution of soil microorganisms in karst tiankeng. Our study narrows this gap by investigating the bacterial biodiversity in different small-scale sites in karst tiankengs. Our research also indicated that the bacterial community’s structure and its distribution also showed a high habitat heterogeneity. For example, Chloroflexi was more abundant in the US, Nitrospirae was more abundant in the BC, and Actinobacteria was more abundant in the OST site. Furthermore, Chlorobi, Spirochaetae, Deinococci-Thermus, and WS6 were specific to the BC, and Deferribacteres, Microgenomates, and BJ-169 were specific to the MS, while Firmicutes and WWE3 were specific to the OST site. The LEfSe analysis further confirmed that the five samples had their own indicator taxa from phylum to genus. The structure of soil bacterial communities (PCoA) also revealed that all samples (except for the ES sample) were well-separated from each other. It is worth noting that the ES site showed the characteristics of differentiation on the inside and outside of the tiankeng in the soil bacterial community structures and the enterotypes. PCoA analysis showed that the ES–3 grouped closer together with all the OST samples, and the enterotype analysis also indicated that the ES–3 and all the outside sites strongly grouped with the OTU1331 (Desulfurellaceae) type, suggesting that there is higher similarity between the bacterial communities of these four sites. This result may contribute to the similarity of soil characteristics and steeper slopes in both the ES3 site and all the outside sites because they have the same origin, resulting in similar contents of TOC, TN, and many other elements (see above). These results suggested that the microhabitats inside of tiankengs may play a critical role in determining the soil bacterial community’s composition and its distribution.

Unlike the plant diversity, the soil bacterial diversity in the Shengmu tiankeng is not affected by the sampling sites. However, there are significant differences between the inside samples and outside samples of the Shengmu tiankeng. These differences suggest that the main controlling factors of the bacterial communities are different from those of the plant communities in karst tiankengs. Previous studies showed that topography characteristics, human disturbances, and moisture are effective predictors for the plant diversity in different tiankengs [16] and the climate (temperature and humidity, with a large range) of cool-adapted plant numbers in dolines [3]. Within dolines, it is not surprising that cooler and more stable microclimates or more nutrient- and moisture-rich habitats may provide more convenient growth conditions for cool-adapted endemic plant taxa [3,34,35]. Our study also showed that there was a significant correlation between the bacterial community richness and the soil moisture and temperature. Previous studies showed that, when the soil is moist and cool, the relative abundance of Acidobacteria is lower [36,37,38]. In contrast, our results indicated that, in the lower soil temperature and higher soil moisture site of the Shenmu tiankengs (such as the BC site), Acidobacteria was more abundant than in the higher soil temperature and lower soil moisture site of the Shenmu tiankeng (such as the ES site) and the site outside of the Shenmu tiankeng (OST site) (Figure A3). Proteobacteria, which was also found to be largely affected by the soil moisture and temperature to a large extent [7], was not affected by any environmental factors in our study. Furthermore, the relative abundance of WS6, Parcubacteria, Chlamydiae, Saccharibacteria, Fibrobacteres, Elusimicrobia, Spirochaetae, FCPU426, and Omnitrophica was positively correlated with the soil moisture. In addition, the relative abundance of WS6 and Deinococci-Thermus, which were specific to the BC site, was negatively correlated with the soil temperature. These results indicated that microclimate factors (e.g., soil temperature and moisture) determined the differential response of the soil bacterial community composition to a small degree in the karst tiankeng.

In addition to the soil temperature and moisture, our study also shows that the soil pH and slope are key factors associated with the bacterial community compositions at the OTU level. Previous studies have shown that the soil pH is considered to be a key factor affecting the soil bacterial community’s composition in many studies of different types of soils [39,40] and across different geographical scales [41,42]. However, little is known about the microbial communities and structure of the karst tiankeng, as a relatively independent special environment [4]. It is possible that our study will greatly expand our understanding of karst tiankeng systems. In general, soil pH was positively correlated with bacterial community compositions in soils [26,43]. However, our results show that, at the phylum level, Chlorobi, WS2, Peregrinibacteria, and Planctomycetes are all negatively correlated with the soil pH of the Shenmu tiankeng, suggesting that the mechanism of the soil pH influence on bacteria may be different from that of other ecosystems [29]. This result also agrees with a previous study indicating that soil pH is negatively correlated with Alphaproteobacteria in a karst cave, which is explained by the increased acid rainfall in the surface soils of the cave [26].

In particular, the slope has been considered an important factor that affects the distribution and number of cool-adapted plant taxa in both tiankengs and dolines [3,5]. Our results show that the lower slope sites, such as the BC, MS, and US sites, could receive more litter, resulting in a higher TOC and TN content, and a lower soil temperature and higher soil moisture resulted in a cool microclimate (Table 1). Our results show that slope is negatively correlated with some bacterial phyla, such as Parcubacteria, Spirochaetae, and Chlamydiae (Figure 4B). Combined with our results, we can draw the conclusion that slope may determine the number and distribution of both plant and bacteria in tiankengs and dolines. The possible reason may be that the gentler slope could not only receive more nutrients but also supply more suitable habitats by maintaining a cooler and more stable microclimate [3,44].

In addition, our results show that some metal elements (such as Ca, Mg, Fe, and Cs) in soil significantly correlated with the bacterial taxa in the Shenmu tiankeng ecosystem (Figure 4B). Previous investigations indicated that heavy metals in soil can alter the microbial community and lead to a change in the microbial community’s structure and a decrease in microbial biomass [44,45,46,47]. For instance, Parcubacteria are negatively correlated with TP, Mg, Fe, and Cs. As a major component of the karst environment and one of the key elements in determining the structure and function of karst ecosystems, Ca has been proven to be one of the dominant factors influencing bacterial composition [48,49,50]. Our results also show that Actinobacteria, Elusimicrobia, and Fibrobacteres are positively correlated with soil Ca, suggesting that these bacteria may dominate the exchange of Ca in the environment of the karst tiankeng system. These results suggest that the soil element content, soil pH, and slope are much more important factors than the topography characteristics or plant diversity in determining the species richness of bacteria in karst tiankeng systems.

## 5. Conclusions

Overall, our results show that, compared with the outside of karst tiankeng, the soil on the inside of a tiankeng could receive more litter and less residue formed after the weathering of carbonate rocks, resulting in higher contents of TOC, TP, and TN but lower contents of many metal elements in these soils. Additionally, our results first reveal that the distribution pattern of the bacterial community diversity in the karst tiankeng system do not match the elevational diversity pattern of plants. Furthermore, our results also first showed the distribution characteristics of bacterial communities in the karst tiankeng systems, which might be controlled by microhabitat characteristics, such as soil moisture, soil temperature, slope, and pH. Given that karst tiankengs serve as modern refugia, with a better capacity to act as safe havens for biodiversity in changing climates and richly diverse indigenous biota, the microbiota can promote the development of plant species under various environmental conditions, and a better understanding of the various interactions taking place at the system level can offer advantages for potential applications in the forest restoration of the degraded karst area.

## Figures and Tables

**Table 1:** Summary of the environment conditions and factor characteristics at each sample site.

Characteristics	BC	MS	US	ES	OST
Shannon–Wiener index	4.25 ± 0.35 ^b^	5.48 ± 0.48 ^a^	3.63 ± 0.77 ^b^	2.10 ± 0.56 ^c^	2.69 ± 0.72 ^c^
Slope (°)	5.33 ± 1.23 ^e^	18.00 ± 2.25 ^c^	11.00 ± 2.11 ^d^	43.00 ± 5.58 ^b^	50.00 ± 14.25 ^a^
Soil moisture (%)	55.44 ± 10.25 ^a^	45.27 ± 12.36 ^ab^	52.74 ± 9.87 ^a^	36.44 ± 7.55 ^b^	32.24 ± 8.33 ^b^
Soil temperature (°C)	12 ± 1.25 ^c^	16 ± 2.22 ^b^	17 ± 1.87 ^a^	15 ± 0.58 ^b^	17 ± 1.14 ^a^
Soil TOC (%)	40.35 ± 10.29 ^a^	38.97 ± 7.66 ^a^	39.73 ± 8.69 ^a^	20.67 ± 7.33 ^b^	13.73 ± 10.22 ^b^
Soil TN (mg/kg)	23.17 ± 4.65 ^a^	23.15 ± 8.12 ^a^	26.33 ± 5.66 ^a^	13.27 ± 4.32 ^b^	10.70 ± 3.18 ^b^
Soil TP (mg/kg)	1.17 ± 0.15 ^b^	1.28 ± 0.25 ^ab^	1.54 ± 0.14 ^a^	1.47 ± 0.22 ^ab^	1.49 ± 0.19 ^a^
Soil pH	6.55 ± 0.32 ^b^	6.85 ± 0.27 ^ab^	7.01 ± 0.15 ^a^	6.97 ± 0.23 ^a^	6.73 ± 0.31 ^ab^
K (g/kg)	5.08 ± 1.98 ^b^	5.93 ± 2.13 ^b^	5.38 ± 3.10 ^b^	9.98 ± 2.88 ^a^	9.74 ± 1.67 ^a^
Ca (g/kg)	29.16 ± 7.22 ^ab^	23.87 ± 8.38 ^bc^	35.65 ± 10.35 ^a^	14.54 ± 3.21 ^c^	2.47 ±1.23 ^d^
Mg (g/kg)	0.51 ± 0.33 ^c^	1.02 ± 0.45 ^bc^	0.74 ± 0.28 ^bc^	1.97 ± 0.77 ^ab^	2.50 ± 1.25 ^a^
Fe (g/kg)	1.91 ± 0.58 ^b^	2.50 ± 0.74 ^b^	2.30 ± 0.98 ^b^	33.37 ± 12.18 ^ab^	74.73 ± 25.33 ^a^

Note: Different lower letters denote a significant difference between the sampling sites (*p* < 0.05). BC, the bottom central part of the Shenmu tiankeng; MS, the middle slope of the Shenmu tiankeng; US, the upper slope of the Shenmu tiankeng; ES, the east slope of the Shenmu tiankeng; OST, the outside of the Shenmu tiankeng; TP, total phosphorus; TN, total nitrogen; TOC, total organic carbon.

**Table 2:** Summary of the Illumina MiSeq sequenced soil bacteria richness and diversity indices (at 97% sequence similarity) for each sample.

Sample	BC	MS	US	ES	OST
Quality sequences	36,890 ± 6275	37,263 ± 2633	38,495 ± 5109	36,453 ± 5072	37,981 ± 5240
Observed OTU	27,796 ± 6852	26,102 ± 5741	26,054 ± 5563	24,217 ± 3875	27,654 ± 4120
Chao rechness	1982 ± 87 ^b^	2175 ± 74 ^a^	2110 ± 69 ^ab^	2033 ± 88 ^b^	2038 ± 103 ^b^
Shannon’s diversity	6.45 ± 1.23	6.47 ± 1.77	6.49 ± 2.31	6.38 ± 0.87	6.39 ± 1.14
Simpson’s diversity	0.003 ± 0.001 ^b^	0.004 ± 0.001 ^ab^	0.004 ± 0.001 ^b^	0.004 ± 0.001 ^ab^	0.004 ± 0.001 ^a^
Sobs index	1947 ± 120 ^b^	2146 ± 98 ^a^	2091 ± 79 ^ab^	2024 ± 130 ^b^	2007 ± 117 ^b^
Ace index	1687 ± 75 ^b^	1848 ± 46 ^a^	1822 ± 77 ^ab^	1732 ± 65 ^b^	1712 ± 38 ^b^

Note: Different lower letters denote a significant difference between the sampling sites (*p* < 0.05), and the abbreviations are described in Table 1.

## Appendix A

**Table A1:** Sample locations and coordinates.

Sampling Site	Altitude (m)	Slope (°)	Plant		Soil Type
			Coverage (%)	Dominant Species	
BC	1205–1213	8–13	93	*Machilus glaucifolia, Acer tonkinense subsp. kwangsiense, Baphicacanthus cusia*	Black humus soil
MS	1260–1283	20–28	95	*Platycarya longipes, M. glaucifolia, Ilex macroarpa*	Black humus soil
US	1309–1325	15–19	80	*Pseudotsuga brevifolia, Elatostema involucratum, Metapanax davidii*	Black humus soil
ES	1239–1276	40–48	48	*M. glaucifolia, Carpinus polyneura, Betula austrosinensis*	Light black powdery clay soil and little humus
OST	1387–1402	45–55	54	*Quercus phillyraeoides, P. longipes, Carex* spp.	Light black powdery clay soil

Abbreviations: BC, the bottom central of the Shenmu tiankeng; MS, the middle slope of the Shenmu tiankeng; US, the upper slope of the Shenmu tiankeng; ES, the east slope of the Shenmu tiankeng; OST, outside of the Shenmu tiankeng.

**Table A2:** Concentrations of major and trace elements in the soil samples of the Shenmu Tiankeng and the outside surface.

**Sample** **Site**	**Li** **(ppm)**	**Be** **(ppb)**	**B** **(ppm)**	**Na** **(ppm)**	**Al** **(ppm)**	**Sc** **(ppm)**	**Ti** **(ppm)**	**Co** **(ppm)**	**Ni** **(ppm)**	**Ga** **(ppm)**	**As** **(ppb)**	**Y** **(ppm)**
BC	0.010	0.067^b^	0.031^b^	0.903^b^	9.833^c^	0.029^b^	2.03^b^	0.008^b^	0.092^b^	0.027^b^	6.08^b^	0.015^b^
MS	0.037	0.129^b^	0.034^b^	1.337^ab^	19.121^bc^	0.029^b^	3.21^b^	0.009^b^	0.105^b^	0.032^b^	7.37^b^	0.028^b^
US	0.011	0.126^b^	0.035^b^	0.978^b^	11.245^c^	0.026^b^	2.15^b^	0.009^b^	0.098^b^	0.024^b^	6.59^b^	0.007^b^
ES	0.068	0.974^a^	0.055^a^	1.451^ab^	59.797^ab^	0.062^a^	14.48^a^	0.053^a^	0.524^a^	0.090^ab^	10.72^ab^	0.075^b^
OST	0.093	1.537^a^	0.053^a^	2.009^a^	99.031^a^	0.077^a^	20.59^a^	0.073^a^	0.853^a^	0.123^a^	14.75^a^	0.205^a^
**Sample** **site**	**Zr** **(ppm)**	**Nb** **(ppm)**	**Pb** **(ppm)**	**Ag** **(ppb)**	**Cd** **(ppm)**	**Sb** **(ppm)**	**Te** **(ppb)**	**Cs** **(ppm)**	**La** **(ppm)**	**Ce** **(ppm)**	**Pr** **(ppm)**	**Nd** **(ppm)**
BC	0.049^b^	0.007^b^	0.001^b^	0.710^c^	0.011^c^	0.016	1.192	0.005^b^	0.010^b^	0.019^b^	0.002^b^	0.015^b^
MS	0.072^b^	0.009^b^	0.002^b^	0.782^bc^	0.011^c^	0.018	1.224	0.008^b^	0.020^b^	0.031^b^	0.004^b^	0.030^b^
US	0.037^b^	0.008^b^	0.001^b^	0.810^bc^	0.014^bc^	0.018	1.054	0.008^b^	0.004^b^	0.009^b^	0.001^b^	0.007^b^
ES	0.473^a^	0.060^a^	0.009^a^	1.664^b^	0.024^b^	0.022	2.073	0.007^b^	0.019^b^	0.062^ab^	0.008^b^	0.070^ab^
OST	0.650^a^	0.076^a^	0.013^a^	2.644^a^	0.039^a^	0.024	2.529	0.018^a^	0.061^a^	0.114^a^	0.021^a^	0.115^a^
**Sample** **site**	**Sm** **(ppm)**	**Eu** **(ppb)**	**Tb** **(ppm)**	**Dy** **(ppm)**	**Ho** **(ppb)**	**Tm** **(ppb)**	**Lu** **(ppb)**	**Ta** **(ppb)**	**W** **(ppb)**	**Tl** **(ppb)**	**U** **(ppb)**	
BC	0.002^b^	0.371^b^	0.231^b^	0.001^b^	0.240^b^	0.098^b^	0.097^b^	0.303^b^	2.758b^c^	0.543^b^	0.644^c^	
MS	0.003^b^	0.667^b^	0.442^b^	0.002^b^	0.412^b^	0.151^b^	0.135^b^	0.442^b^	4.949^bc^	0.794^b^	0.742^c^	
US	0.001^b^	0.236^b^	0.093^b^	0.001^b^	0.118^b^	0.047^b^	0.042^b^	0.420^b^	2.240^c^	0.793^b^	1.063^bc^	
ES	0.008^b^	1.962^b^	1.323^b^	0.008^b^	1.626^b^	0.788^b^	0.711^b^	3.171^a^	7.130^ab^	1.386^a^	2.734^ab^	
OST	0.019^a^	4.193^a^	3.011^a^	0.018^a^	3.814^a^	1.754^a^	1.651^a^	4.889^a^	10.236^a^	1.538^a^	3.335^a^	

Different lower letters denote a significant difference between the sampling sites (*p* < 0.05), tested by One-Way ANOVA (*p* < 0.05). Abbreviations are described in Table A1.

**Table A3:** Mantel test of GeoChip data with environmental attributes.

**Environmental Factors**	**Moisture**	**PH**	**Soil Temperature**	**Plant Shannon–Wiener**		**TP**	**TOC**
*p*	0.014	0.007	0.012	0.977		0.528	0.079
*r*	0.473	0.589	0.476	0.0041		0.103	0.374
**Environmental factors**	**TN**	**Slope**	**Ca**	**Mg**	**Fe**	**K**	**Cs**
*p*	0.103	0.001	0.186	0.874	0.09	0.315	0.072
*r*	0.336	0.607	0.263	0.029	0.336	−0.163	0.520

Abbreviations: TP, total phosphorus; TOC, total organic carbon; TN, total nitrogen; Shan., plant Shannon–Wiener; Cs, Caesium; K, Potassium; Mg, Magnesium; Ca, Calcium; Fe, Ferrum.
